# Attenuated dengue viruses are genetically more diverse than their respective wild-type parents

**DOI:** 10.1038/s41541-021-00340-5

**Published:** 2021-05-20

**Authors:** Amanda Makha Bifani, Milly M. Choy, Hwee Cheng Tan, Eng Eong Ooi

**Affiliations:** 1 Programme in Emerging Infectious Diseases, Duke-NUS Medical School, Singapore, Singapore; 2 Viral Research and Experimental Medicine Centre, SingHealth Duke-NUS Academic Medical Centre, Singapore, Singapore; 3 Saw Swee Hock School of Public Health, National University of Singapore, Singapore, Singapore

**Keywords:** Microbiology, Vaccines, Virology

## Abstract

Dengue poses a significant burden of individual health, health systems and the economy in dengue endemic regions. As such, dengue vaccine development has been an active area of research. Previous studies selected attenuated vaccine candidates based on plaque size. However, these candidates led to mixed safety outcome in clinical trials, suggesting it is insufficiently informative as an indicator of dengue virus (DENV) attenuation. In this study, we examined the genome diversity of wild-type DENVs and their attenuated derivatives developed by Mahidol University and tested in phase 1 clinical trials. We found that the attenuated DENVs, in particular the strain under clinical development by Takeda Vaccines, DENV2 PDK53, showed significantly higher genome diversity than its wild-type parent, DENV2 16681. The determinant of genomic diversity was intrinsic to the PDK53 genome as infectious clone of PDK53 showed greater genomic diversity after a single in vitro passage compared to 16681 infectious clone. Similar trends were observed with attenuated DENV1 and DENV4, both of which were shown to be attenuated clinically, but not DENV3 that was not adequately attenuated clinically. Taken together, evidence presented here suggests that genome diversity could be developed into a marker of DENV attenuation.

## Introduction

Dengue is the most common mosquito-borne viral diseases globally. An estimated 100 million people around the world develop acute dengue annually, some of whom progress to life-threatening severe dengue^1^. Dengue is caused by any one of four antigenically distinct dengue viruses (DENV1-4). As the geographic footprints of both DENVs and their principal mosquito vector, *Aedes aegypti* expand from the tropics to the subtropics, the number of people living in at risk regions is set to increase to over 6.1 billion people by 2080^2^. These trends underscore the urgency for an effective dengue vaccine to protect those living and traveling to at risk areas.

A tetravalent dengue vaccine is needed to protect against all four DENVs. Acute infection with one DENV would trigger durable adaptive immunity against the homologous but not to the heterologous DENVs^3^. Moreover, immunity from primary DENV infection places individuals at risk of severe dengue from an antibody-enhanced secondary infection with a heterologous DENV^4,5^. Attempts to develop a full genome-based live attenuated DENVs have had mixed outcome. In particular, an international collaboration led by Mahidol University successfully produced the DENV2 PDK53 strain^6^, which has been shown to possess good safety and efficacy profiles in clinical trials^7^. However, the DENV3 derivative, PGMK30/FRhL3 produced using the same in vitro and in vivo criteria for attenuated strain selection, induced dengue-like illness among vaccinees^8^. This mixed outcome underlines the need for a more informative indicator of DENV attenuation to avoid unnecessary failures in clinical development.

Studies on yellow fever virus (YFV) has found that genome diversity may be an indicator of viral fitness and hence attenuation^9–11^. The attenuated YFV strain, YF17D has a significantly lower genome diversity compared to its parental wild-type Asibi strain^9^. Likewise, the French neurotropic vaccine (FNV) strain of yellow fever, which was derived through serial mouse brain passage of the French viscerotropic virus (FVV), showed similarly reduced genome diversity compared to its wild-type parent^10^. However, while FNV was successfully used to reduce the burden of YFV in francophone Africa, it has since been discontinued as a vaccine due to high rates of post-vaccination encephalitis in children^12^. If indeed genome diversity could inform on the clinical fitness of flaviviruses, then deep sequencing of viral genomes could be a simple approach to identifying attenuated strains for further development as vaccine candidates.

Here, using deep sequencing, we compared genome diversity of wild-type DENVs and their respective attenuated derivatives, developed by Mahidol University and tested in a phase I clinical trial^13^. We found that, unlike YFV, DENV2 PDK53 has significantly more genome diversity than its parental wild-type strain, DENV2 16681. The increased genome diversity observed in PDK53 compared to wild-type 16681 was also observed in infectious clones of these viruses, suggesting that increased genome diversity is an intrinsic phenotype of PDK53. We also found that in addition to PDK53, the clinically attenuated DENV1 PDK13 and DENV4 PDK48 strains also showed significantly more genome diversity than their parental wild-type strains. Remarkably, no significant difference in genome diversity was observed between the under-attenuated DENV3 PGMK-30/FRhL-3 and the wild-type DENV3 from which it was derived. Altogether, our data show that in contrast to YF17D, attenuated DENVs are more genetically diverse than wild-type strains^14^.

## Results

### PDK53 is more genetically diverse than wild-type strain 16681

**Fig. 1:**
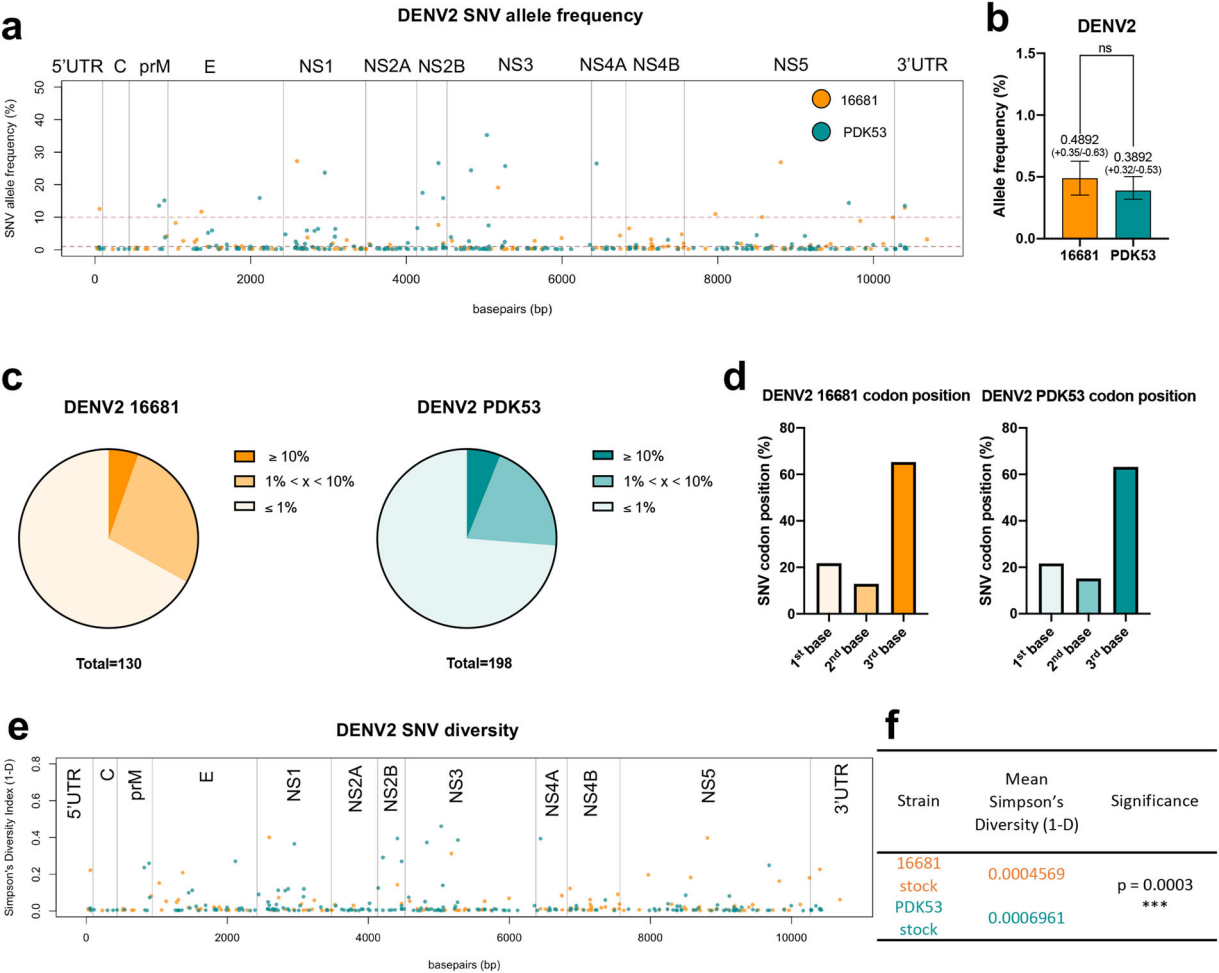
DENV2 attenuated strain PDK53 is more genetically diverse than its parent wild-type strain 16681. **a** The allele frequency of SNVs identified in DENV2 wild-type 16681 (orange, *n* = 130) and its attenuated derivative PDK53 (teal, *n* = 198), are plotted along the genome. Horizontal dashed lines indicate an allele frequency of 1% (bottom) and 10% (top). **b** The mean allele frequency of SNVs identified in DENV2 16681 and PDK53 with their respective 95% confidence intervals. Significance was assessed by Mann–Whitney *U* test. **c** The percentage of SNVs with an allele frequency of ≤1, 1 < x < 10, or ≥10% in DENV2 16681 and PDK53. **d** The percentage of SNVs that locate to the 1st, 2nd, or 3rd codon position in both DENV2 16681 and PDK53. **e** The diversity of SNVs at each position in DENV2 16681 (orange) and PDK53 (teal) as measured by Simpson’s diversity index. **f** The mean diversity of each SNV in DENV2 16681 (orange) and PDK53 (teal). Significance was assessed by Mann–Whitney *U* test.

We first performed high throughput sequencing on wild-type DENV2 16681 and its attenuated derivative, PDK53. PDK53 differs from 16681 by nine consensus mutations, of which 3 are non-synonymous mutations (Supplementary Fig. 1a). To distinguish variants from the average sequencing error rate, we used the program Lofreq, which identifies single nucleotide polymorphisms by incorporating base-call quality scores as error probabilities into its model and assigns a *p*-value to each variant^15,16^. Lofreq was used to identify lower frequency allele variants that would otherwise be missed using conventional variant callers. Overall, our deep sequencing data showed positional variance that were evenly distributed throughout the genomes of both DENV2 strains (Fig. 1a). We found a greater number of SNVs in PDK53 (*n* = 198) than 16681 (*n* = 130) (Fig. 1a). However, the mean SNV allele frequency was less than one percent for both strains and was not significantly different between the two DENV2 strains (Fig. 1b). The low mean allele frequency observed is consistent with the observation that the majority of single nucleotide variants (SNVs) occurred at a frequency of less than or equal to 1% (Fig. 1c). These low frequency variants that make up a large portion of the SNVs population found, would have been dismissed as sequencing error using variant calling packages other than Lofreq.

The majority of these SNVs were also located in the third base position of the codons, which is least likely to result in an amino acid change (Fig. 1d). As a result most SNVs were silent (Supplementary Table 1). There were also considerably more transitions than transversions in both DENV2 strains (Supplementary Fig. 1b, c). Only 5.4% of SNVs in 16681 and 6.1% in PDK53 were above 10% in allele frequency (Fig. 1a, c). None of the SNVs with an allele frequency of above 10% resulted in stop codons, suggesting that these SNVs did not produce defective viral genomes (DVGs). Furthermore, when stocks of PDK53 and 16681 culture supernatants were analyzed for DVGs using DI-tector^17^, no DVGs were observed for either virus after reads matching human or viral genomes were filtered out.

We next compared the whole genome diversity using Simpson’s diversity index (Fig. 1e, f). Simpson’s diversity index reflects the abundance of each of the four nucleotides at a given site in the genome. The diversity at each SNV site on the genomes of 16681 and PDK53 is shown in Fig. 1e. Non-parametric Mann–Whitney test found that the Simpson’s diversity index of PDK53 was significantly higher than that of 16681 (Fig. 1f). This same trend was also observed when another measure of genome diversity, Shannon entropy (H’) was used (Supplementary Fig. 1d, e). H’ measures the uncertainty in predicting the identity of a base pair when it is randomly selected from the population; the greater the uncertainty, the higher the diversity at that site in the genome.

### DENV2 infectious clones recapitulate the increased diversity of PDK53

Our analyses thus far made use of DENVs that were isolated from patients and subsequently passaged numerous times in vitro. As a result, through multiple rounds of replication, these viral stocks would likely have acquired SNVs to form a heterogenous population. To determine if the observed differences in SNV diversity was due to passage history or factors intrinsic to the viral genomes, we repeated the above analysis using infectious clones of 16681 and PDK53. Infectious clones offered the opportunity to achieve a more homogenous starting point with the same passage history for the two genomes, thereby excluding virus passage history as a confounding factor. Infectious clones of DENV2 16681 and PDK53 were constructed based on previously reported full genome sequences of these strains using the Gibson assembly method as previously described^13^. Both viruses were then rescued in HEK293T cells (P0) and propagated once in *Aedes albopictus*-derived C6/36 cells (P1) and deep sequenced.

**Fig. 2:**
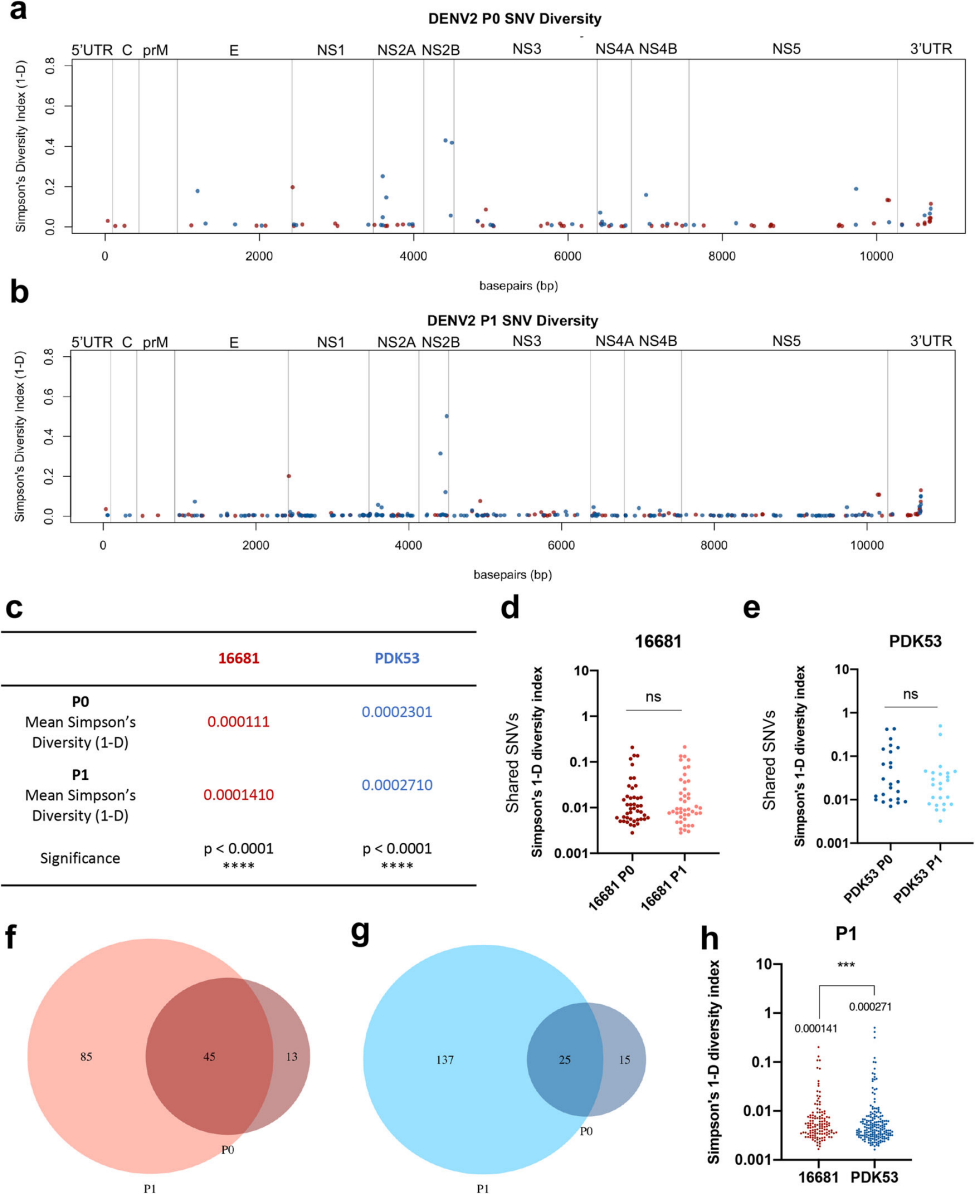
DENV2 PDK53 infectious clone is more genetically diverse than DENV2 16681 infectious clone. Allele diversity measured by Simpson’s index at variant positions along the DENV2 genome of 16681 (red) and PDK53 (blue) at (**a**) passage 0 (P0) and (**b**) passage 1 (P1). **c** Simpson’s diversity of SNVs from 16681 and PDK53 infectious clones at P0 and P1. A Mann–Whitney *U* test was used to determine significance. Diversity of overlapping SNV sites between P0 and P1 in (**d**) 16681 and (**e**) PDK53. Statistical significance was determined using Mann–Whitney *U* test. Venn diagram of overlapping and novel SNV positions in DENV2 (**f**) 16681 and (**g**) PDK53 between P0 and P1. **h** Whole genome diversity of DENV2 16681 (red) and PDK53 (blue) infectious clone at P1. The mean diversity is shown above the respective data set. Statistical analysis between the two genomes was performed using Mann–Whitney *U* test.

As expected, deep sequencing analysis of the infectious clones found fewer SNVs than cultured viruses (Supplementary Table 2). The genome diversities of both 16681 and PDK53 infectious clones were significantly increased after a single passage (16681 *p* < *0.0001;* PDK53 *p* < *0.0001*) (Fig. 2a–c). To examine whether diversity after passage resulted from the acquisition of novel SNVs or selection of existing SNVs, we compared the diversity of the SNVs shared between P0 and P1 for 16681, as well as PDK53. If the SNVs found in both P0 were undergoing selection at P1, then the allele frequency of shared SNVs between P0 and P1 would be expected to increase, resulting in an increase in diversity at these sites. However, shared SNVs showed no significant difference in diversity for either 16681 (Fig. 2d) or PDK53 (Fig. 2e), suggesting that that increased diversity after passage arose from the acquisition of novel SNVs, and not selection of existing SNVs.

We also found that PDK53 acquired more novel SNVs at P1 (*n* = 137) than 16681 (*n* = 85) (Fig. 2f, g). Consistent with this, DENV2 PDK53 had significantly more genome diversity than 16681 after passage when measured using Simpson’s diversity index (Fig. 2h). Analysis using Shannon entropy (H’) also showed the same result (Supplementary Fig. 2a–d). Notably, despite the significantly elevated genetic diversity of PDK53 compared to 16681, variants were generally not detected in the attenuating mutation sites of PDK53, suggesting that the stability of the attenuated mutations were maintained (Table 1). The exception was a T to A variant at position 5270, which was located in the NS3 gene in the PDK53 stock (Table 1). This variant has been observed previously and is consistent with the literature^7^. A number of SNVs observed in the DENV2 infections clones were also observed in the stock virus (Supplementary Fig. 2e–f). Overall, the trend of increased diversity of the attenuated PDK53 strain compared to the wild-type 16681 strain was reproducible using infectious clones. This finding suggests that the extent of genome diversity is influenced by factors intrinsic to the DENV2 genome.

**Table 1:** Allele frequency at the 9 nucleotide positions that differentiate DENV2 16681 from PDK53.

Position	Reference allele	Allele frequency (%)			Alternate allele	Allele frequency (%)		
		Stock	P0	P1		Stock	P0	P1
**16681**								
57	C	100%	100%	100%	U	0%	0%	0%
524	A	100%	100%	100%	U	0%	0%	0%
2055	C	100%	100%	100%	U	0%	0%	0%
2579	G	100%	100%	100%	A	0%	0%	0%
4018	C	100%	100%	100%	U	0%	0%	0%
5270	A	100%	100%	100%	U	0%	0%	0%
5547	U	100%	100%	100%	C	0%	0%	0%
6599	G	100%	100%	100%	C	0%	0%	0%
8571	C	89.03%	100%	100%	U	10.97%	0%	0%
**PDK53**								
57	U	99.23%	100%	99.7%	C	0.77%	0%	0.30%
524	U	100%	100%	100%	A	0%	0%	0%
2055	U	100%	100%	100%	C	0%	0%	0%
2579	A	97.47%	100%	99.61%	G	2.53%	0%	0.39%
4018	U	97.87%	100%	99.86%	C	2.13%	0%	0.14%
5270	U	74.3%	100%	100%	A	25.70%	0%	0%
5547	C	100%	100%	100%	U	0%	0%	0%
6599	C	100%	100%	100%	G	0%	0%	0%
8571	U	100%	100%	100%	C	0%	0%	0%

### Greater genetic diversity in DENV1 PDK13 and DENV4 PDK48 than their wild-type parental strains

**Fig. 3:**
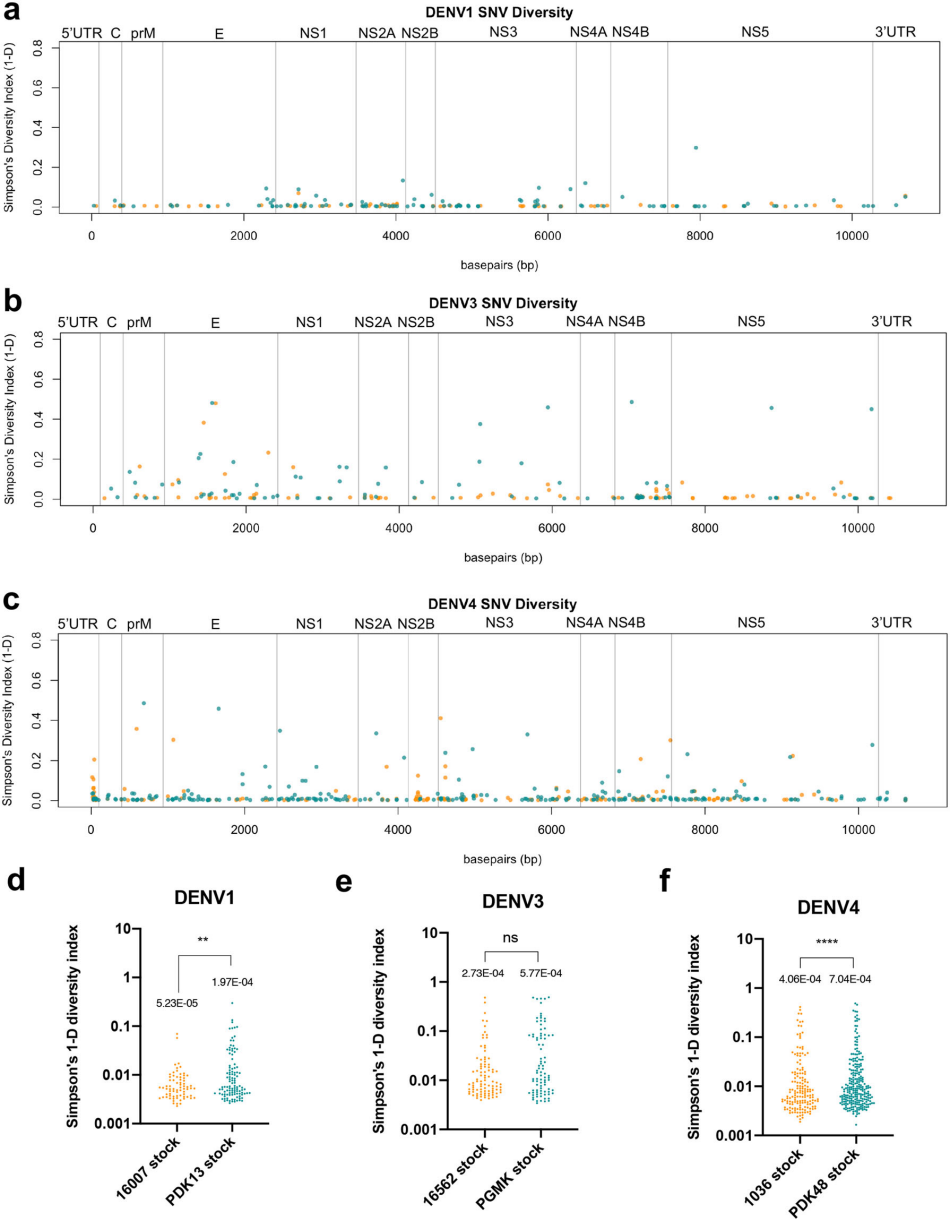
DENV attenuated strains are genetically more diverse than their parent wild-type strains. Diversity of SNVs, measured by Simpson’s diversity index, identified in (**a**) DENV1 (**b**) DENV3, and (**c**) DENV4 wild-type isolate in orange and vaccine candidates in teal. The mean diversity of each SNV in (**d**) DENV1, (**e**) DENV3, and (**f**) DENV4 wild-type isolates (orange) and vaccine candidates (teal). Significance was analyzed by Mann–Whitney *U* test.

We next asked if increased genomic diversity also occurred in the other attenuated DENVs developed under the Mahidol University led effort^6,18^. We compared the genome diversity of live attenuated vaccine (LAV) candidates, DENV1 PDK13 and DENV4 PDK48 with their respective wild-type parents, DENV1 16007 and DENV4 1038. We also contrasted these comparisons with an analysis between the genome diversity of DENV3 16562 and its under-attenuated derivative, DENV3 PGMK30/FRhL3. SNVs were identified throughout the genome and plotted along the genome of their respective serotypes (Supplementary Fig. 3a). No change in the consensus mutations that differentiated wild-type serotypes from their derived vaccine candidates (Supplementary Fig. 3b) was found. Positional variance was observed throughout the genome of all three serotypes of DENVs (Fig. 3a–c). Similar to PDK53, Simpson’s diversity of clinically attenuated DENV1 PDK13 and DENV4 PDK48 were significantly elevated compared to their respective wild-type parents (Fig. 3d–f). In contrast, the under-attenuated PGMK30/FRhL3 showed no significant difference in genome diversity compared to its wild-type DENV3 16562 parent (Fig. 3d–f). The same trends were also observed when Shannon’s entropy was used to assess genome diversity (Supplementary Fig. 3d).

## Discussion

Previous endeavors to derive attenuated strains for each of the four DENVs used plaque size as a primary selection factor. While this method identified attenuated DENV1, 2 and 4 strains in the form of PDK13, PDK53 and PDK48, respectively, DENV3 PGMK30/FRhL3 retained a virulent phenotype^6^. Inadequately attenuated DENVs were also found in other attempts to derive dengue vaccine candidates^6,19^. Furthermore, when YFV was attenuated through serial passaged in HeLa cells, the plaques got larger^20^. A more reliable method for ascertaining attenuation would be useful to avoid costly failures in clinical trials.

Genome diversity has previously been shown to contribute to changes in viral fitness and pathogenesis^14^. Due to the error prone nature of their replication machinery, the RNA dependent RNA polymerase (RdRp) enzyme, RNA viruses exist as a collection of closely related genomes, or quasispecies. RdRp has an error rate of 1 in 10^4^, which translates approximately to one mutation per genome in virus with 10 kb genome^21^. Genomic diversity enables the viruses to adapt to novel ecological niches, including avoidance of population bottlenecks^22^. Indeed, previous works suggest that an optimal amount of genetic diversity is required for viral fitness and deviation from this may result in an attenuated phenotype^14^.

On one side of the spectrum, too little diversity has been associated with restricted tropism, such as those of hepatitis C virus (HCV)^23^, chikungunya virus^24^, poliovirus^14^ as well attenuated yellow fever virus (YFV)^9^; reduced variant population limit virus dissemination to different cell types within the host organism^22^. On the other side of the spectrum, high diversity has also been associated with reduced viral fitness^25–28^; too much diversity could lead to fewer viable viruses with increased proportion of variants bearing premature stop codon, copy-back or other catastrophic mutations^29^. Keeping the genomic diversity within a limited range may be critical for viruses to sustain fitness.

Here, we found that successfully attenuated DENV1, 2, and 4 strains - PDK13, PDK53 and PDK48 - have increased genome diversity compared to the wild-type clinical isolates from which they were derived. In contrast, the DENV3 vaccine candidate, PGMK30/FRhL-3, which failed to show adequate safety in clinical trials, had no significant difference in genome diversity when compared with the wild-type DENV3 16562. Remarkably, the infectious clone of DENV2 PDK53 showed increased diversity after only a single passage compared to that of wild-type 16681, further supporting the notion that extent of genome diversity could be encoded in the genome. The increase in diversity between passage zero and one was due to several novel low frequency variants rather than at sites shared between the two passages. This finding suggests that new variants which emerge in the population could be more beneficial than selecting for pre-existing SNVs. This is in line with recent studies which proposed that virus strains with high genome diversity could serve as candidates for live attenuated vaccines^25,26,28^.

**Fig. 4:**
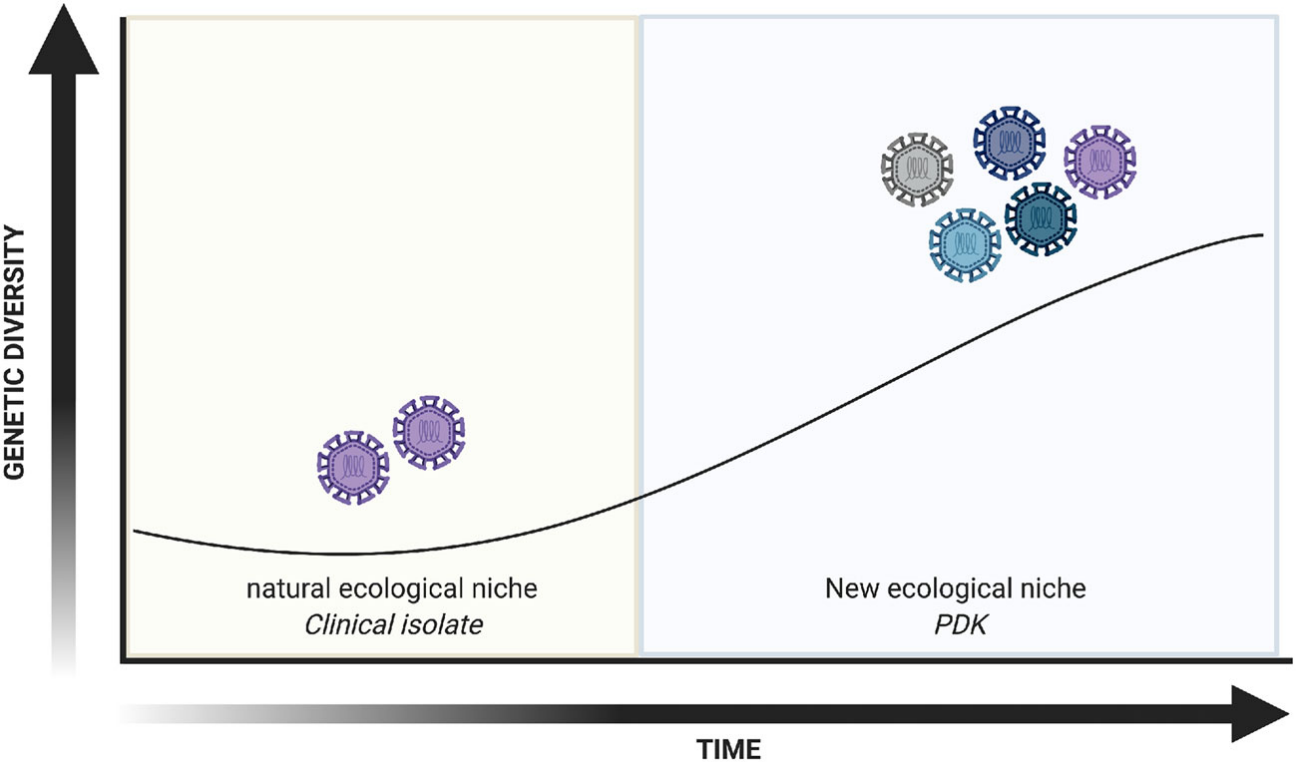
Hypothetical diagram of changes in dengue genome diversity. As dengue circulates in its natural ecological niche, the virus undergoes selection pressures imposed by the alternating human host and mosquito vector. When the virus is transferred to a new ecological niche in vitro and continually passaged, the past selective pressures of host and vector are replaced with those of the new niche. Images are created using Biorender.

While the mechanisms contributing to increased genome diversity in attenuated dengue remains unknown, it is possible that the increase in diversity of attenuated strains is a generalized result of dengue serial passage. Despite the low fidelity of its RdRp, the genome diversity of DENV remains limited in nature due to its continual transition between humans and mosquitoes - two hosts with very different immune environmental bottlenecks^15^. Here, we propose that increased genetic diversity in a novel niche could serve as a fitness advantage to select variant viruses that are best suited for a new environment (Fig. 4). This is best exemplified by the increase in novel SNVs in DENV2 16681 and PDK53 infectious clones when they were transferred from their initial passage in HEK293T to C6/36 cells that served as a new environment at passage one. Furthermore, the attenuated DENV1, 2 and 4 strains from Mahidol University were derived from viruses passaged on primary dog kidney (PDK) cells and selected for their small plaque phenotype^6,30^. It is possible that upon initial viral passage in the new PDK niche, the genetic diversity of the virus increased, providing a greater pool of variants in the absence of an immune barrier to create a bottleneck on genome diversity. These strains thus have the potential to serve as vaccine candidates as they have adapted to a novel ecological niche and were thus attenuated when placed back into its natural niche in humans and mosquitoes.

Conversely, the wild-type DENV3 16562 failed to form plaques in PDK cells, possibly as a result of limited diversity in its genome to cope with a novel environment. DENV3 16562 could not be expanded in PDK cells and were thus passaged in primary green monkey kidney cells (PGMK) 30 times and fetal rhesus lung cells three times to derive the PGMK30/FRhL3 strain. Both of these cell types originated from non-human primates, which are more closely related to humans than dogs. These cells may not provide the novel environmental niche needed to generate genomic diversity for the discovery of a genetic variant that would be attenuated in humans and mosquitoes. This possibility is also supported by the lack of genetic diversity observed in Zika virus after passage in Vero (African green monkey kidney epithelial) cell-line^31^.

Notably, our data supports the notion that deviation from an optimal level of genome diversity could affect viral fitness^14^. However, the mechanism of attenuation of DENV is different to those that operate to attenuate YF17D which has less genome diversity compared to the wild-type strain^9,10,23^. The YF17D strain has a mutation in the non-structural 5 (NS5) protein which encodes the RdRp that leads to increased fidelity during virus replication^32^ and consequently mitigates genome diversity^9^. Conversely, the Mahidol University DENV vaccine candidates do not harbor non-synonymous mutations in their RdRp, implying that increased diversity observed is likely independent of RdRp mutations. However, mutations outside of the RdRp have been known to affect replication fidelity^33^. Increased genome diversity has been linked to production of DVGs^29^. While DVGs have been observed in circulating dengue strains^34^, no DVGs were found in stock used in this study. However, as our sequencing was performed on virus from supernatant, it is possible that DVGs do result from increased diversity within the cell but are not packaged inside the exiting virion and thus are not picked up in our analysis.

In conclusion, compared to their wild-type strains, our study shows an association between successfully attenuated dengue vaccine strains and increased whole genome diversity. This trend can be replicated in an infectious clone derived DENV and after only a single passage. Our findings suggest that divergence in genome diversity from the parental wild-type strain as a potential marker of attenuation, which may serve useful in the discovery of second and third generation dengue vaccine candidates.

## Methods

### Cell cultures and dengue viruses

BHK-21 (ATCC CCL-10), C6/36 (ATCC CRL-1660) cells were purchased from the American Type Culture Collection (ATCC). BHK-21 and C6/36 cells were cultured in RPMI 1640 supplemented with 9% fetal calf serum (FCS). Maintenance media used for infections contains 2% FCS, 25 mM Hepes, 100 U/ml penicillin, and 100 µg/ml streptomycin.

DENV strains of the original Mahidol stocks (DENV1 16007, DENV1 PDK13, DENV2 16681, DENV2 PDK53, DENV3 16562, DENV3 PGMK30FRhL3, DENV4 1036 and DENV-4 PDK48) were obtained from Dr Claire Huang (Centers for Disease Control and Prevention, USA) and were passaged three to five times in C6/36 cells in our laboratory.

### Generation of infectious clones

Viral genome RNA was extracted from laboratory stocks of DENV2 16681 and PDK53. cDNA was synthesized with SuperScript^®^ III First-Strand Synthesis Kit as per manufacturer’s instructions. Mutant viruses were constructed using Gibson assembly^35^. Viral genome RNA was extracted from laboratory stocks of DENV2 16681 and PDK53. Six PCR fragments of around 2000 nucleotides were generated from cDNA using six primer pairs. These fragments were gel-purified with MinElute^®^ Gel Extraction Kit and TA cloning was performed into pGEM^®^-T Easy Vector. Six viral fragments were amplified from the vectors using Q5^®^ Hot-Start High-Fidelity 2x Master Mix. Vector similar to what was previously described was also amplified using primer pairs^36^. Amplified fragments were then gel-purified and equimolar amounts (0.1 pmole) of each genome fragment and the amplified vector were assembled using NEBuilder^®^ HiFi DNA Assembly Master Mix at 50 °C for 60 min to generate infectious clones. Infectious clones were transfected into HEK293T cells using Lipofectamine^®^ 2000 Reagent as per manufacturer’s instructions. Supernatants containing viruses were harvested 3 days after transfection.

### Infection of C6/36 cells

DENV2 16681 and PDK53 were passaged once on C6/36 cells. Briefly, C6/36 cells were infected with each virus (MOI = 0.1) and cell-free supernatant were harvested at 6 days post-infection. Infections were performed in triplicates for each virus. The input viruses, and supernatant from infected C6/36 cells were sent for next generation sequencing for variance analyses. The average sequencing depth of samples is specified in Supplementary Table 3.

### Next generation sequencing

Viral genomic RNA was isolated from DENV1, 2, 3 and 4 supernatant using QIAamp Viral RNA Mini Kit without carrier RNA. Library prep was completed using the NEBNext Ultra II Directional RNA Library Prep Kit for Illumina and sequencing was carried out on an Illumina^®^ MiSeq™ system. Reads were trimmed and assessed for quality using Cutadapt 2.8 and FastQC respectively. Reads were aligned to reference sequences DENV2 16681 (NC001471.2), DENV2 PDK53 (KU725664.1), DENV3 (KU725665/1), DENV1 (AF180818.1) and DENV4 (AY618989.1) using an in house pipeline using the bowtie package^37^. The infection triplicates were pooled and aligned to the same reference genome for variant calling rather than averaging the individual replicates. Variant allele frequency was determined using the lofreq package which is more sensitive to low frequency allele variants than conventional variant calling programs^38^.

### Determining presence of DVGs

The presence or absence of DVGs was performed using the DI-tector package in python^17^. Breifly, DI-tector identifies four classes of DVGs (DVGs with (i) several internal deletions, (ii) complex defective interfering genomes, (iii) 5’ snap-back or (iv) 5’ copy-back) by aligning trimmed fastq files to the host and viral genome in search of unmatched reads that could possibly be DVGs. The remaining reads are segmented, and the fragments are realigned to the viral genome in search of matching fragments corresponding to DVGs. Matches are then filtered and counted. The reference host genome used was GCF_000001405.37_GRCh38.p11_genomic.

### Calculating genome diversity

Genome diversity was calculated using Simpson’s (1-D) diversity index as well as Shannon entropy (H’) following the equations listed below. Diversity was calculated at each position using the allele frequency of the four bases. Whole genome statistical analysis was performed using all sites in the genome, including the majority of which had no diversity present. Mann–Whitney *U* test was used to determine significance. However, for easy visualization of allele variant diversity, the diversity values that were 0 were removed.

1 $$Simpson^\prime s\,1 - D\,diversity\,index\,D = 1 - \left( {\begin{array}{*{20}{c}} {{\sum} {n\left( {n - 1} \right)} } \\ {N\left( {N - 1} \right)} \end{array}} \right)$$

2 $$Shannon\,entropy\,H^\prime = - \mathop {\sum}\limits_{i = 1}^R {pi\ln pi}$$

### Reporting summary

Further information on research design is available in the Nature Research Reporting Summary linked to this article.

## Supplementary information

Reporting Summary

Supplementary files

## Data Availability

Variant call files from the lofreq analysis have been deposited on the European Variation Archive (EVA) at https://www.ebi.ac.uk/ena/browser/view/PRJEB43929. Data relevant to this study is also available from the corresponding author, E.E.O., upon requestion.
