# Supplementary material for: Attenuated dengue viruses are genetically more diverse than their respective wild-type parents
Source: NPJ Vaccines. 2021 May 20;6:76. doi: 10.1038/s41541-021-00340-5 (PMC8138019; doi:10.1038/s41541-021-00340-5)
Supplement: Supplementary file 2 — Supplementary files [file 41541_2021_340_MOESM2_ESM.pdf]

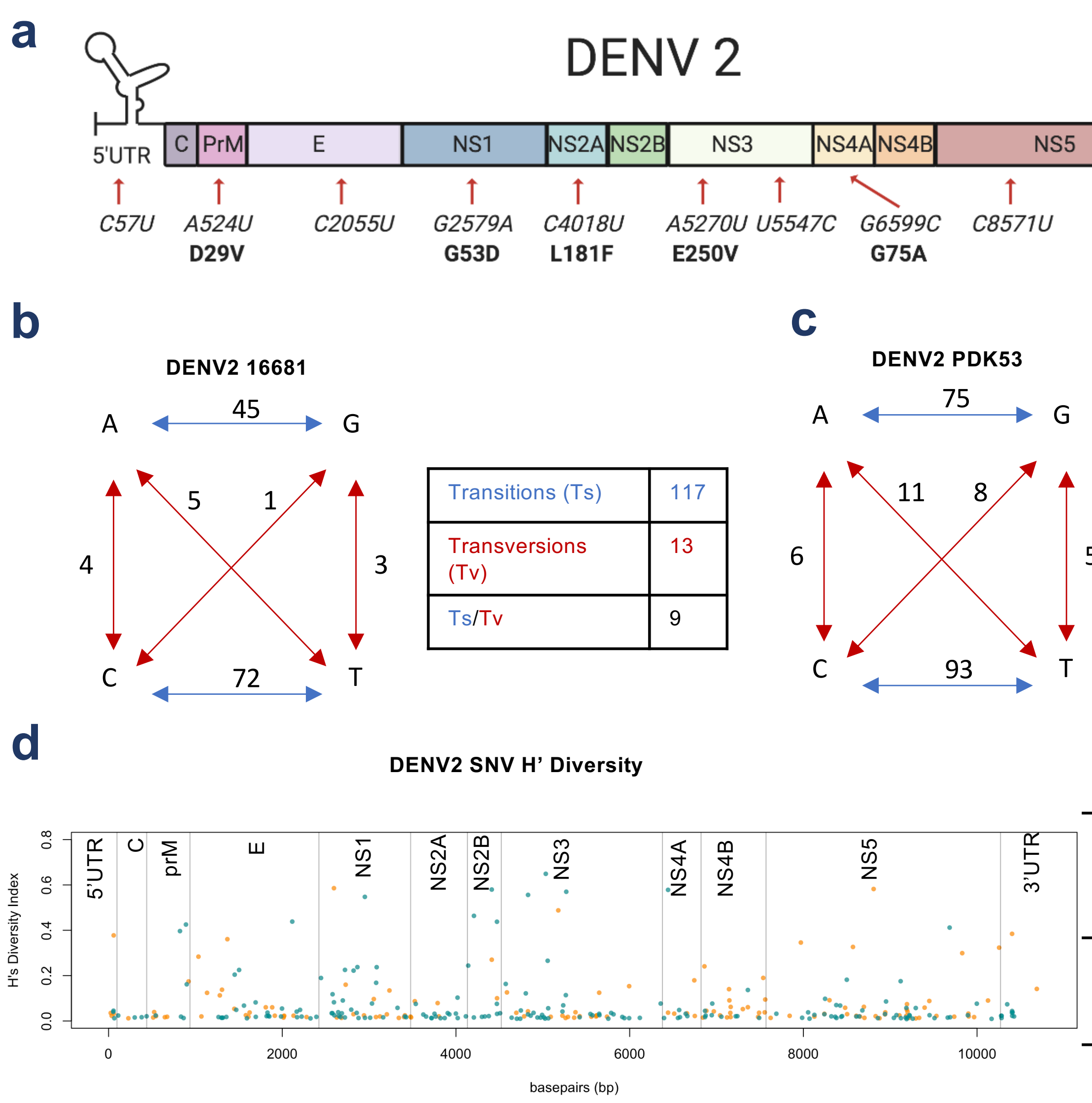

e

Strain	Mean Shannon Entropy (H')	Significance
16681 stock	0.0009538	p = 0.0003 ***
PDK53 stock	0.001336	

Supplementary Figure 1. DENV2 PDK53 has elevated Shannon's entropy than DENV2 16681. a) Schematic diagram of the DENV2 genome. Arrows indicating the mutations that occurred in 16681 to become PDK53. Nucleotide changes are indicated in italics while amino acid differences are shown in bold below the nucleotide position. Image created with Biorender. (b-c) The proportion of SNVs that resulted from transitions (blue) and transversions (red) in (b) 16681 and (c) PDK53. (d) Diversity, measured by Shannon's entropy depicted along the DENV2 genome for 16681 (orange) and PDK53 (teal). (e) The mean Shannon's entropy of each DENV2 genome. Significance was calculated using a Mann-Whitney U test.

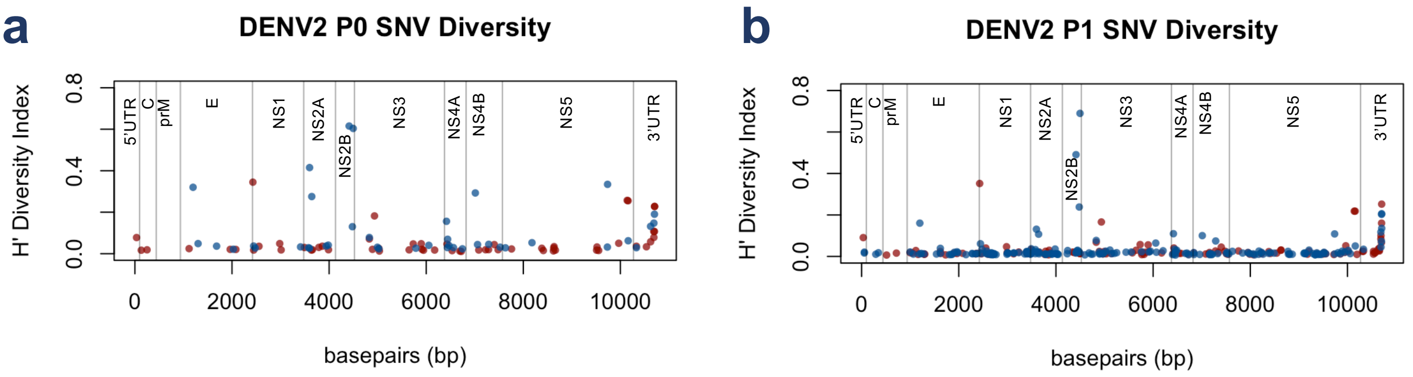

c

	16681	PDK53
**P0** Shannon Entropy	0.0002930	0.0004358
**P1** Shannon Entropy	0.0003684	0.0006087
Significance	p < 0.0001 ****	p < 0.0001 ****

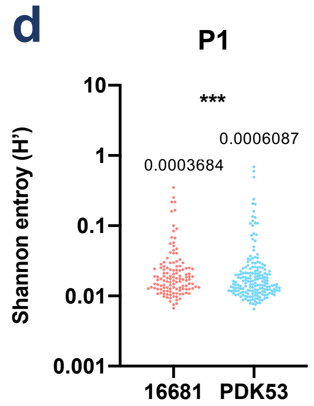

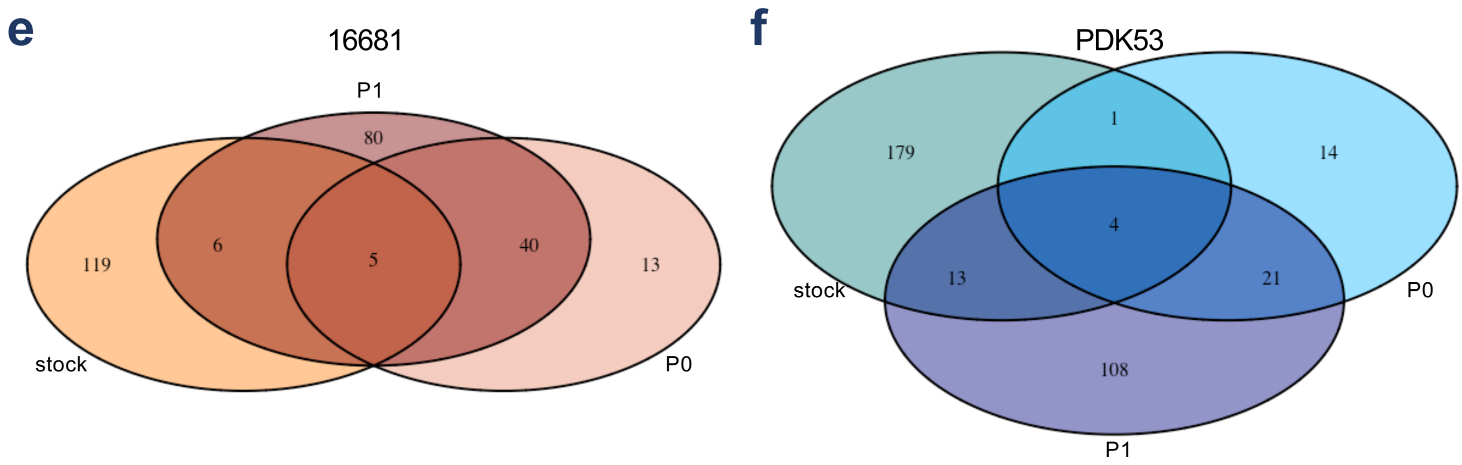

Supplementary Figure 2. DENV2 PDK53 infectious clone has elevated Shannon's entropy than DENV2 16681 infectious clone. (a-b) Shannon's entropy as a measurement of diversity at variant positions along the DENV2 genome of 16681 (red) and PDK53 (blue) at (a) passage 1 (P1) and (b) passage 0 (P0). (c) Mean genome diversity of DENV2 infectious clones at P0 and P1 of 16681 and PDK53. The whole genome diversity was assessed for significance by Mann Whitney U test. (d) Graphical representation of the SNV genome diversity of DENV2 16681 (red) and PDK53 (blue) infectious clones at P1. Diversity was statistically compared by Mann Whitney U test. (e-f) Venn diagram showing overlapping SNVs found in the stock virus, the infectious clone at P0 and at P1 for DENV1 16681(e) and PDK53(f).

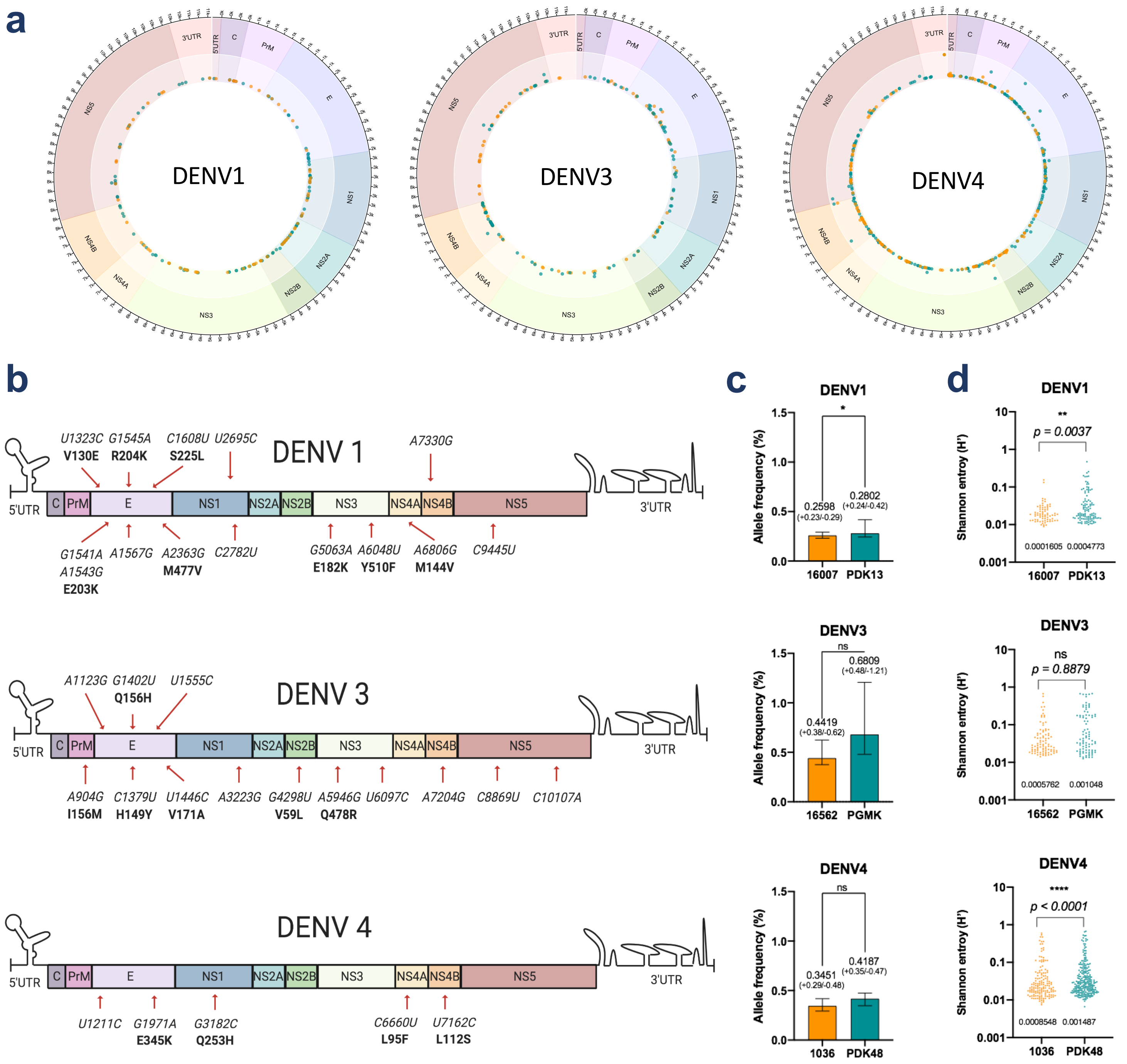

Supplementary Figure 3. Genetic diversity following serial passage of DENV1, 3 and 4 serotypes. (a) Circos plots of DENV1, 3 and 4 wild-type and vaccine strains. The outer track shows the genome and position, while the inner track shows the allele frequency of any alternate alleles for the wild-type strain (orange) and vaccine strain (teal) (b) Schematic diagram of DENV1, 3 and 4 genomes structure. Red arrows indicate the position of mutations (nucleotide changes are indicated in italics; amino acid difference are in bold). Images were created with Biorender. (c) The median allele frequency of SNVs in DENV1, 3 and 4. Statistical analysis was performed using Mann-Whitney U test. (d) SNV diversity in DENV1, 3 and 4 measured by Shannon's entropy. Significance was determined by Mann-Whitney U test.

Supplementary Table 1: Table depicting the SNVs with an allele frequency above 10% in (a) 16681 and (b) PDK53.

a

DENV2 16681

Position	Ref allele	SNV allele	Amino acid substitution	Gene
**59**	T	C	NA	5'UTR
**1368**	C	T	silent	E
**2595**	C	T	silent	NS1
**5178**	A	G	silent	NS3
**7971**	C	T	silent	NS5
**8571**	C	T	silent	NS5
**8809**	A	G	silent	NS5

b

DENV2 PDK53

Position	Ref allele	SNV allele	Amino acid substitution	Gene
**822**	G	C	L > F	prM
**891**	A	G	silent	prM
**2115**	G	A	silent	E
**2951**	T	C	V > A	NS1
**4207**	A	G	I > V	NS2B
**4412**	C	T	T > I	NS2B
**4830**	A	G	silent	NS3
**5034**	T	C	silent	NS3
**5270**	T	A	V > E	NS3
**6442**	A	G	N > D	NS2B
**9684**	A	G	silent	NS5
**10403**	T	C	NA	3'UTR

Supplementary Table 2. The number of SNVs identified in the DENV2 stock and infectious clones at P0 and P1 in C6/36 cells. The number of variants with an allele frequency above 1% are indicated in brackets.

Virus	Stock SNVs (> 1% AF)	P0 SNVs (> 1% AF)	P1 SNVs (> 1% AF)
16681	130 (43)	57 (13)	125 (11)
PDK53	198 (52)	40 (16)	184 (18)

Supplementary Table 3. Average read coverage per nucleotide position.

Strain	Serotype	Average coverage per nucleotide (standard deviation)
PDK13 stock	Dengue 1	7402.98 ± 743.90
16007 stock		7752.59 ± 757.40
PDK53 stock	Dengue 2	7778.52 ± 719.36
PDK53 P0 infectious clone		3646.18 ± 787.12
PDK53 P1 infectious clone		7871.92 ± 652.48
16681 stock		7740.27 ± 804.92
16681 P0 infectious clone		7736.79 ± 823.92
16681 P1 infectious clone		7861.20 ± 621.50
PGMK stock	Dengue 3	7693.97 ± 900.54
16562 stock		7620.78 ± 850.96
PDK48 stock	Dengue 4	7820.62 ± 557.21
1036		7807.54 ± 587.38
